# Local Enrichment with Convergence of Enriched T-Cell Clones Are Hallmarks of Effective Peptide Vaccination against B16 Melanoma

**DOI:** 10.3390/vaccines12040345

**Published:** 2024-03-22

**Authors:** Anna Vyacheslavovna Izosimova, Alexandra Valerievna Shabalkina, Mikhail Yurevich Myshkin, Elizaveta Viktorovna Shurganova, Daria Sergeevna Myalik, Ekaterina Olegovna Ryzhichenko, Alina Faritovna Samitova, Ekaterina Vladimirovna Barsova, Irina Aleksandrovna Shagina, Olga Vladimirovna Britanova, Diana Vladimirovna Yuzhakova, George Vladimirovich Sharonov

**Affiliations:** 1 Research Institute of Experimental Oncology and Biomedical Technologies, Privolzhsky Research Medical University, Nizhny Novgorod 603950, Russia; izosimova_a@pimunn.net (A.V.I.); liza2309shur@yandex.ru (E.V.S.); zavyalova_d@pimunn.net (D.S.M.); yuzhakova-diana@mail.ru (D.V.Y.); 2 Institute of Translational Medicine, Pirogov Russian National Research Medical University, Moscow 117997, Russia; shabalkina_av@rsmu.ru (A.V.S.); ryzhichenko.e@phystech.edu (E.O.R.); barsova_ev@rsmu.ru (E.V.B.); shagina_ia@rsmu.ru (I.A.S.); britanova_ov@rsmu.ru (O.V.B.); 3 Department of Genomics of Adaptive Immunity, Shemyakin-Ovchinnikov Institute of Bioorganic Chemistry RAS, Moscow 117997, Russia; mikhailmyshkin.ibch@gmail.com; 4 Pathoanatomical Department, Nizhny Novgorod Regional Clinical Cancer Hospital, Nizhny Novgorod 603126, Russia; 5 Center for Precision Genome Editing and Genetic Technologies for Biomedicine, Pirogov Russian National Research Medical University, Moscow 117997, Russia; alinasamitova16@gmail.com

**Keywords:** peptide anticancer vaccine, B16 melanoma, TCR repertoire

## Abstract

Background: Some peptide anticancer vaccines elicit a strong T-cell memory response but fail to suppress tumor growth. To gain insight into tumor resistance, we compared two peptide vaccines, p20 and p30, against B16 melanoma, with both exhibiting good in vitro T-cell responses but different tumor suppression abilities. Methods: We compared activation markers and repertoires of T-lymphocytes from tumor-draining (dLN) and non-draining (ndLN) lymph nodes for the two peptide vaccines. Results: We showed that the p30 vaccine had better tumor control as opposed to p20. p20 vaccine induced better in vitro T-cell responsiveness but failed to suppress tumor growth. Efficient antitumor vaccination is associated with a higher clonality of cytotoxic T-cells (CTLs) in dLNs compared with ndLNs and the convergence of most of the enriched clones. With the inefficient p20 vaccine, the most expanded and converged were clones of the bystander T-cells without an LN preference. Conclusions: Here, we show that the clonality and convergence of the T-cell response are the hallmarks of efficient antitumor vaccination. The high individual and methodological dependencies of these parameters can be avoided by comparing dLNs and ndLNs.

## 1. Introduction

Vaccination with mRNA-encoded antigens is a powerful immunological approach that was significantly boosted during the recent COVID-19 pandemic. Prior to the pandemic, it had been under extensive development for various malignancies, including cancer. In work by the group of Ugur Sahin, who was one of the leading developers of Pfizer’s SARS-CoV-2 vaccine [1], initially, this technology had been developed as a cure for B16 melanoma [2,3]. Shortly after studies in mice and just before the pandemic, it had undergone trials as therapeutics for patients with metastatic melanoma and glioblastoma [4,5]. For metastatic melanoma, this trial demonstrated a 75% remission rate for over 2 years.

Several B16 peptide vaccines are also efficient in the induction of mutation-reactive cytokine-secreting T-cells and in tumor control [2,3]. Peptide vaccines are promising therapeutic agents since they are easier to produce and store compared to mRNA vaccines. However, in spite of their good T-cell induction properties, peptide vaccines often exhibit modest tumor control [6,7]. There are multiple factors that skew T-cells’ responses in certain directions, including reaction to the adjuvant, organ-/tissue-/cell-type targeting, peptide length, TCR affinity, pharmacokinetics, and similarity with autoantigens [8,9]. The complexity of such factors hinders the prediction of the integral response and requires testing in each individual case.

To evaluate the mechanism underlying the efficiency of peptide vaccines, we used neoantigen peptide vaccines, described previously for B16 murine melanoma [2]. The top 50 mutations were selected according to their expression, MHC binding, and functional impact. Then, 27mer peptides with each of these mutations were evaluated for immunogenicity in vitro in comparison with their wild-type counterparts. We selected two of these peptides that demonstrated a maximal response to the mutated epitope with no response to the WT variants. Their epitopes are within *Tubb3* (MUT20: G402A, peptide p20) and *Kif18b* (MUT30: K739N, peptide p30). MUT30 appeared to be the most efficient B16 antitumor vaccine in both the peptide and mRNA forms, while data on MUT20’s effect on tumor growth are lacking [2,3].

Here, we compared two B16 peptide neoantigen vaccines, p20 and p30, discovered in [2]. We observed that the induction of T-cell responsiveness to the neoantigens does not correlate with antitumor activity. Via the in vitro restimulation of cells from vaccinated mice with antigen peptides, we showed that both vaccines elicit T-cell reactivity, but more prominently for p20. However, in the p20-vaccinated mice, the T-cells had a much lower tumor reactivity compared with the p30-vaccinated mice, which correlated with effects on tumor growth. By comparing cytotoxic T lymphocyte (CTL) repertoires, we showed that after p30 vaccination, the CTL clonality increased in the tumor-draining lymph nodes (dLNs), and enriched clones had a branch of similar T-cell receptor (TCR) variants. This is contrary to that of p20, which induced none of these features. This observation supports the consensus that clonality and convergence of the antigen response are key factors in effective antitumor immunity.

## 2. Materials and Methods

### 2.1. Mice Vaccination and Tumor Model

The experiments were carried out on transgenic C57Bl/6-FoxP3-EGFP mice (kindly provided by Alexander Rudensky, Sloan Kettering Institute, New York, NY, USA). The transgenic mice were bred against a C57Bl/6 genetic background by knocking in the chimeric construct of the eGPF subcloned into the first exon of the FoxP3 gene [10].

The mice were vaccinated with synthetic peptides p20 (FRRKAFLHWYTGEAMDEMEFTEAESNM) and p30 (PSKPSFQEFVDWENVSPELNSTDQPFL), both from Genscript Biotech B.V. (Rijswijk, The Netherlands). 40 mg/mL peptide stock solutions were prepared in DMSO (Sigma-Aldrich Co. LLC, St. Louis, MO, USA) and stored at −20 °C. Vaccinations were administered 21 and 7 days before lymphocyte isolation or tumor inoculation. Each vaccination was performed with 50 µg of the peptide per mouse in PolyI:C or complete Freund adjuvant (CFA) (both from InvivoGen Inc., San Diego, CA, USA). For the CFA injections, the peptides were dissolved in PBS at 500 µg/mL and mixed thoroughly with CFA at a 1:1 ratio. A total of 200 µL of peptide/CFA emulsion was injected subcutaneously (s.c.) into each flank and into the back at the tail’s base on both sides. For the PolyI:C vaccination, a total of 200 µL of PBS solution with peptide at 250 µg/mL and PolyI:C at 250 µg/mL was injected s.c. into both flanks.

Tumors were generated by a subcutaneous (s.c.) injection of 5 × 10^4^ B16F0 cancer cells in 300 μL of PBS into the left flank. B16F0 melanoma cells were obtained from the ATCC, expanded, and stored aliquoted at −150 °C. Before the inoculation, the cells were thawed and grown for 2 weeks in DMEM medium (Paneco Ltd., Moscow, Russia) supplemented with 10% fetal bovine serum (FBS, Gibco BRL, Grand Island, NY, USA), 0.06% L-glutamine, 50 units/mL penicillin, and 50 μg/mL streptomycin. The cells were incubated at 37 °C and 5% CO_2_ and passaged 2–3 times per week. Right before the injection, the cells were detached by trypsin, counted, and resuspended at a final concentration of 10^6^ cells in 6 mL of PBS.

### 2.2. Tissue Processing and In Vitro Restimulation

For evaluation of immune memory, the mice were sacrificed at day 0 with isoflurane (Esteve Pharmaceuticals S.R.L., Milan, Italy), and inguinal lymph nodes were isolated. For in vitro restimulation assays, the lymph nodes were minced several times and placed in a 6-well plate in full RPMI medium with glutamine (Life Technologies Ltd., Paisley, UK) supplemented with 10% defined FBS (USA origin, Sigma-Aldrich Co. LLC, St. Louis, MO, USA), 50 units/mL penicillin, and 50 μg/mL streptomycin. After 4 h of outgrowth, the lymphocytes were collected, passed through a 100 μm cell strainer, washed two times with PBS, and stained with 0.5 μg/mL TagIt-Violet (BioLegend Inc., San Diego, CA, USA) for 10 min at 37 °C. After staining with TagIt-Violet, the cells were washed, resuspended in full RPMI medium, and seeded in a 96-well plate in six wells per mouse at 4–5 × 10^5^ cells/well. Either 40 μg/mL of p20 peptide or p30 peptide or an equal amount of DMSO were supplemented in two wells for each mouse. After 7 days of culture, the cells were collected by triple washing with Versene solution (Paneco Ltd., Moscow, Russia), washed, and stained for flow cytometric analysis.

For the study of immunity after tumor challenge, the mice were sacrificed on day 16 of tumor growth. The lymph nodes were isolated and thoroughly minced, passed through a 100 μm cell strainer, washed twice, and processed for analysis by flow cytometry and sorting.

### 2.3. Flow Cytometric Analysis

For staining of cultured cells, the pellets were resuspended in 50 μL RPMI medium with the addition of 1 μL of antibodies per sample: CD4-V450 or CD4-BV650 (both from BD Biosciences, San Jose, CA, USA), CD3-APC or IA/IE-Alexa649 (BioLegend Inc., San Diego, CA, USA), CD69-BV510 (BD Biosciences), CD25-BV605 (BioLegend), and CD8-APC/Cy7 (BD Biosciences). Freshly isolated cells were first stained with biotin-labeled B220/CD45R antibodies (1 μL in 50 μL of RPMI per sample) for 1 h on ice, then washed and stained with the panel of labeled antibodies supplemented with 0.5 μL of Steptavidin-PE (Jackson Immunoreseach Europe Ltd., Ely, UK). The cells were stained for 1.5–2 h in ice, diluted with 200 μL of RPMI, and analyzed/sorted without wash. Flow cytometry and cell sorting were performed on a FACSAriaIII cell sorter (BD Biosciences) equipped with 405 nm, 488 nm, 561 nm, and 633 nm lasers using a 70 μm nozzle. For RNA isolation and repertoire analysis, the cells were sorted directly in 100 μL of RLT lysis and storage buffer (Qiagen GmbH, Hilden, Germany).

The T-cell subset composition and the expression of activation markers were obtained from flow cytometric data using FlowJo v.10 software (BD Biosciences). Percentages of T-cell subsets were analyzed and depicted with GraphPad Prism v. 8 software (GraphPad Software Inc., La Jolla, CA, USA). The data were presented as individual points with mean values. A two-way ANOVA with Šídák’s multiple comparison tests was used for statistical inference.

### 2.4. TCR Library Preparation and Repertoire Sequencing

Total RNA was extracted from RLT solutions using the HiPure Total RNA Kit (Magen Biotechnology Co., Ltd., Guangzhou, China). Next, TCRβ cDNA was obtained from 20–40 ng of eluted RNA by 30 min amplification at 42 °C with primer for TRBV constant region and QScribe III revertase (Gene-quest LLC, Moscow, Russia). The resulting cDNA samples (20 μL) were purified with MagPure A4 magnetic beads (Magen Biotechnology) at a 1:1.5 sample:beads ratio (v:v) and resuspended in 20 μL of 10 mM TE buffer (10 mM Tris/HCL, 1 mM EDTA, pH 8.5). Half of the cDNA was used for multiplex PCR (30 cycles) with forward TRBV-specific primers [11] and TRBC-specific reverse primers. All primers were synthesized by Evrogen (Moscow, Russia). Amplicons were purified using MagPure A4 beads at a 1:1.6 ratio, washed twice with ethanol, resuspended in 50 μL of TE buffer, quantified, and used at 100 ng for library preparation. The libraries were prepared with the MGIeasy FS Library Prep Set (MGI Tech Co., Ltd., Shenzhen, China) according to the manufacturer protocol, starting at the “end repair and A-tailing” stage. The circularized libraries were converted to nanoballs and sequenced with the DNBSEQ-G400 sequencer (MGI) using paired-end 125 + 125 nt reads and about 25 reads per sorted cell. TCR repertoires were extracted from FASTQ reads using MiXCR v.4.5.0. software [12].

### 2.5. TCR Repertoire Analysis

The repertoires were processed using VDJTools v.1.2.1 [13]. For CTL repertoires, only clones with a frequency above 2.5 × 10^−5^ were taken, with a rescaling of the frequencies of the remaining clonotypes to give 100% in total. This frequency threshold was chosen so that the total TCR reads divided by the number of reads in the smallest clone (supposed to be the product of a single cell) were below the number of cells in the sample. Diversity statistics and V-segment usage were calculated with the VDJTools.

To identify vaccine-specific clones, all samples for each vaccine were pooled, and clonotypes with cumulative frequencies above 5 × 10^−5^ were considered further. Unique clonesets were obtained by excluding overlapped clonotypes present in both vaccines. Such overlapped clonotypes constituted 2.1% and 2% within the p20 and p30 groups, respectively.

For the construction of clusters of convergent clonotypes, we used the ALICE algorithm with a random TCRβ background generated with the OLGA model [14,15]. The algorithm was applied to pooled clonesets for each vaccine group using the TCRgraper R library with default parameters for mouse TCRs (https://github.com/KseniaMIPT/tcrgrapher, accessed on 7 March 2024). The clonotype was classified as ALICE hit if it had significantly increased numbers of neighbors (adjusted Benjamini-Hochberg *p* value below 5 × 10^−5^). The resulting ALICE clonesets were clustered with the repseq Python library (https://github.com/mmjmike/repseq, accessed on 7 March 2024) either with or without linking to V and J segments. The clusters were exported, visualized, and analyzed with Cytoscape 3.10.1 (https://cytoscape.org/, accessed on 7 March 2024). Consensus amino acid sequences for the clusters were generated using the online WebLogo tool v.2.8.2 (https://weblogo.berkeley.edu/, accessed on 7 March 2024).

Overlapping of the current clonesets with known antigen-specific TCRs was performed with an R script. Each antigen-specific CDR3 was aligned with the current repertoires, allowing a single amino acid mismatch and/or one indel and exact match to the TRBV of a specific TCR. Alignment was performed by the Biostrings package from the Bioconductor project (https://www.bioconductor.org/, accessed on 7 March 2024). 

## 3. Results

### 3.1. Pre-Vaccination with the p20 Peptide Stimulates Tumor Growth

First, we checked the expression and the presence of the targeted mutation in B16F0 tumor cells by analyzing RNA-seq data from [16]. Both mutations were present in the B16F0 line: MUT30 (*Kif18b*) was encountered in 54% of reads (80 from 148), while MUT20 (*tubb3*) was encountered in 24% of reads (193 from 815). The expression of *tubb3* on the mRNA level appeared to be several times higher than that of *Kif18b* (Figure S1).

**Figure 1:**
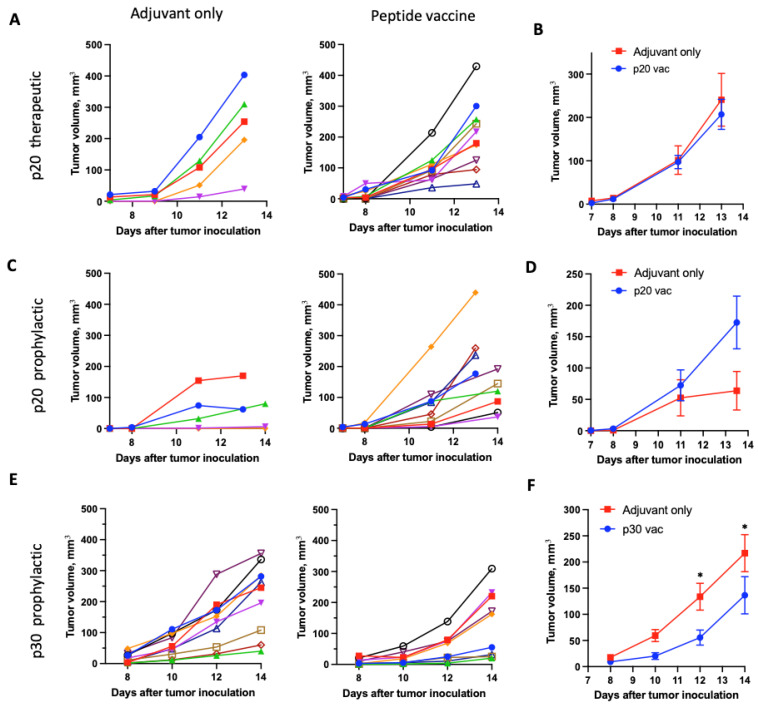
Tumor growth curves of the p20- (**A**–**D**) and p30-vaccinated (**E**,**F**) mice compared to the mice vaccinated only with adjuvant. Curves for individual mice in different colors (**A**,**C**,**E**) and curves for mean ± SEM tumor volume for each group (**B**,**D**,**F**) are shown. Therapeutic vaccination at days 0 and 7 after tumor inoculation was used for the p20 vaccine (**A**,**B**). The prophylactic vaccination scheme 21 and 7 days before tumor inoculation was used for p20 (**C**,**D**) and p30 (**E**,**F**). Tumor volumes on days 13 and 14 were combined as day 13.5 on panel B. (* *p* < 0.05).

Then, we tested the effects of the p20 vaccination on tumor growth, which was not presented in the original papers [2]. As in the corresponding study, we have used PolyI:C adjuvant but with a twice-lower vaccination dose. We vaccinated mice with 50 µg of peptide and 50 µg of PolyI:C s.c. into both flanks. Therapeutic vaccination on days 0 and 7 after tumor inoculation did not affect tumor growth (Figure 1A,B). To enhance vaccination impact, we used a prophylactic vaccination scheme with two doses of vaccine: 3 weeks and 1 week prior to inoculation of tumor cells, both with PolyI:C. Following this scheme, the antitumor activity of p20 did not increase (Figure 1A). Contrary to p20, the prophylactic vaccination with p30 peptide by the same scheme suppressed tumor growth (Figure 1B).

### 3.2. T-Cells from p20-Vaccinated Mice Are More Responsive to In Vitro Restimulation Compared to p30

**Figure 2:**
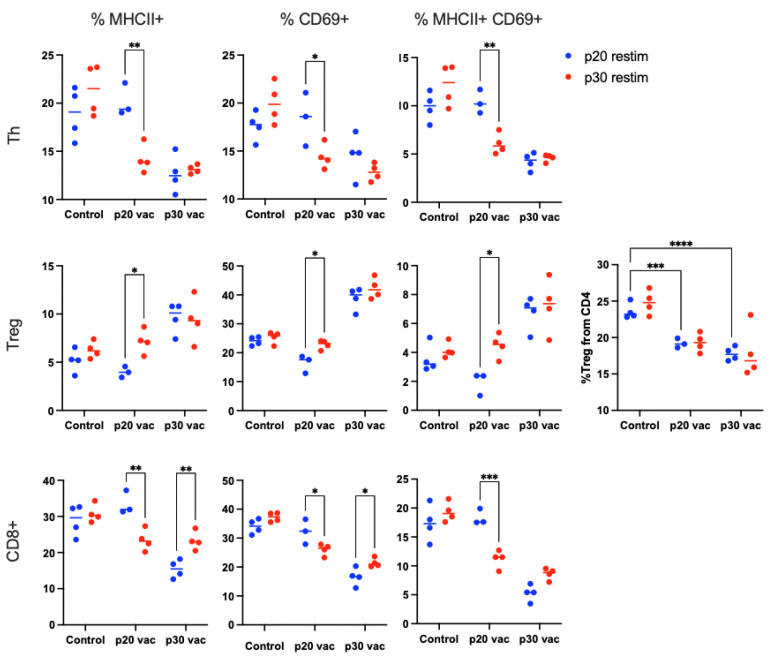
Flow cytometric analysis of T-cell response after restimulation of lymph node derived cells from mice vaccinated with either peptide. The cells were isolated from adjuvant-only (Control), p20-vaccinated (p20 vac), or p30-vaccinated (p30 vac) mice and were restimulated with p20 (blue) or p30 (red) peptides. The relative number of cells expressing either MHCII, CD69, or both activation markers on each cell subset. The percentage of Treg from CD4+ cells is also shown. Statistically significant differences within each vaccinated group are labeled with asterisks (* *p* < 0.05; ** *p* < 0.005; *** *p* < 0.0005, **** *p* < 5 × 10^−5^). Intergroup differences are analyzed and marked only for Treg percentage. The gating scheme used for the dissection of Th, Treg, and CTL subsets and the evaluation of their activation markers is presented in Figure S2.

To evaluate the mechanisms underlying the differences in vaccine activity, we have examined T-cell responsiveness in vitro after vaccination and in vitro restimulation with the antigenic peptides. To extend the duration of antigen exposure during prophylactic vaccination, we have formulated peptides in complete Freund adjuvant (CFA) for the first dosage and PolyI:C for the boosting dose. The cells from mice vaccinated with either of two peptides or adjuvant were isolated and cultured for 7 days in the presence of 40 µg/mL of either p20 or p30 peptides. After cultivation, the cells were analyzed for activation markers with flow cytometry (Figure 2).

Restimulation with both peptides activated CD8+ T-cells from mice vaccinated with the corresponding peptide (Figure 2). p20 but not p30 also stimulated the Th response and decreased the Treg fraction. This data indicate that the p20 vaccine induced T-cell responsiveness to cognate stimuli. Furthermore, this inflammatory in vitro response did not provide antitumor immunity by p20 vaccination.

### 3.3. p30 Vaccine Provides Better Th Tumor Response and Treg Suppression In Vivo

**Figure 3:**
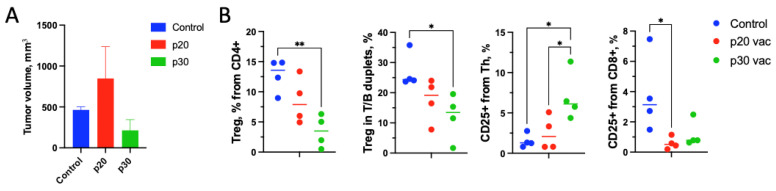
Analysis of T-cell response to tumor after vaccination with adjuvant only (Control), p20 peptide (p20 vac), or p30 peptide (p30 vac). (**A**) Mean tumor volumes for vaccination groups. (**B**) Percentages of T-cell subsets and their activated phenotypes. Only subsets and phenotypes with significant differences between groups are present. (* *p* < 0.05; ** *p* < 0.005). The gating scheme used for dissection of Th, Treg, and CTL subsets and evaluation of CD69 and CD25 activation markers is presented in Figure S2.

To understand the contradictory in vitro and in vivo effects of the p20 vaccine, we analyzed T-cell responses to tumors in mice after p20 or p30 prophylactic vaccination. The mice were vaccinated with peptides in CFA adjuvant 21 days before tumor inoculation and with a boosting dose in PolyI:C adjuvant 7 days before. The vaccinated mice were inoculated with 5 × 10^5^ B16F0 tumor cells at day 0. On day 14, the mice were sacrificed, and the inguinal tumor-draining lymph node (dLN) from the tumor flank and the non-draining lymph node from the opposite flank (ndLN) were isolated. The cells were stained for T-cell subsets together with activation markers CD25 and CD69 and sorted for further TCR repertoire analysis (Section 3.4 and Section 3.5). In these experiments, we have also noticed the opposite effects of the peptides on tumor growth: suppression by the p30 vaccine and promotion by the p20 vaccine (Figure 3A).

We found no difference in lymphocyte subset composition and activation markers between dLN and ndLN and combined them for the following analyses, unless otherwise indicated. We revealed that after the vaccination, the relative number of Treg cells decreased (Figure 3B). This effect was noted for both peptides but was significant only for p30. Among activation markers, CD69 did not reveal any differences, yet the amount of CD25+ T-cells changed after vaccination in a peptide-dependent manner. p30 but not p20 increased CD25+ Th cells significantly compared to the control (adjuvant only), indicating promotion of the Th response by vaccination, which agrees with the previous study [3]. In contrast to Th cells, CD25 expression on cytotoxic CD8+ T-cells decreased after vaccination for both peptides but was more significant for p20.

### 3.4. p30 but Not p20: Vaccine Elicits Clonal Site-Specific Tumor Response

**Figure 4:**
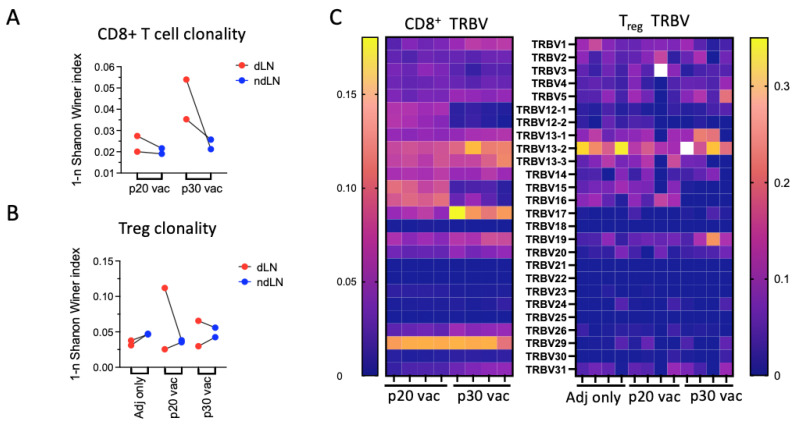
TCR repertoire metrics for adjuvant-only (Adj only), p20-vaccinated (p20 vac), or p30-vaccinated (p30 vac) groups after tumor challenge. (**A**,**B**) Clonality metrics are expressed as [1-normalized Shannon Wiener] [18] of CTL (**A**) and Tregs (**B**) for either vaccinated group for dLNs (red) and nLNs (blue). (**C**) TRBV segment usage in CTL (left) and Treg (right) repertoires for different vaccination groups.

For the evaluation of the TCR repertoire fingerprint of an effective peptide vaccine, we compared the repertoires of sorted CD8+ lymphocytes. Each sorted sample consisted of 7.5 × 10^4^ sorted CD8+ T-cells. Moreover, 1–1.4 × 10^6^ on target TCR reads and 1.4–1.44 × 10^4^ clonotypes were pooled for each sample. We compared dLN with ndLN according to the notion that tumor-specific clones are enriched in dLNs [17]. We have found sharp differences in dLN/ndLN clonality between the CTL repertoires of p20- and p30-vaccinated mice. For p30-vaccinated mice, TCR were significantly more clonal in dLN compared to ndLN, while for p20, this difference was modest (Figure 4A). Vβ segment usage in the repertoires has also demonstrated significant differences between p20 and p30-vaccinated mice, indicating vaccine-specific responses to tumor challenge (Figure 4C).

For dissection of vaccine-specific clonotypes, we have combined truncated repertoires for each vaccine by CDR3 amino acid (see Section 2.5). After that, the clonotypes present both in the p20 and p30 groups were removed, resulting in two repertoires of unique clonotypes. The top five clonotypes for each repertoire and their frequencies in each sample are presented in Table 1. It is notable that for p20, just the last one from the top five clones was found in both mice, while four of the top five clonotypes from p30 were found in both mice, all with prevalence in the dLN over the ndLN. The search for these clonotypes through the available data revealed that two p30-enriched clonotypes were found earlier in relevant studies. Clonotypes with CASSPGQANTEVFF and CASSDRVEQYF CDR3s were found in anti-CTLA-4-responsive mice with MC38 colon cancer and during response to allogeneic skin graft, respectively [19,20]. A convergent variant of the top dLN-enriched clonotype, CASSRDNYAEQFF, was found within anti-CTLA-4-induced clonotypes and among public TCR variants [19,21].

One of the reasons for the weaker in vivo tumor response in the case of p20 may be the induction of Treg cells by vaccination [25,26,27]. To check whether the vaccine stipulates Tregs, we compared the Treg repertoires in the p20, p30, and “adjuvant only” groups. Among 12 samples, only one had outlying clonality, and it was within the p20 group (Figure 4B). This does not exclude that Treg induction may be the possible reason for the limited p20 in vivo efficiency. However, limited statistics and the depth of Treg repertoires do not allow us to make any distinct statements.

### 3.5. Major dLN Expanded Clones in p30 but Not in p20 Have Clusters of Convergent Variants

Commonly, epitope-specific responses result in a bunch of similar TCRs with shared sequence features that represent a cluster of neighboring (by similarity) TCRs [28,29]. For the identification of such antigen-specific responses, we analyzed the clustering of TCRβ clonotypes for each vaccine. For these purposes, we joined four repertoires for each peptide and applied the ALICE clustering algorithm using a random TCRβ repertoire generated with the OLGA model as the reference [15,16].

**Figure 5:**
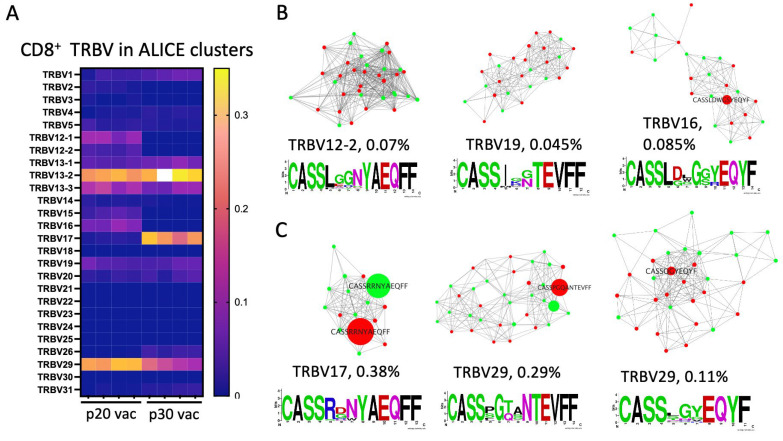
Analysis of clonotypes within ALICE clusters. (**A**) TRBV segment usage in ALICE clusters of CTLs from p20 and p30 vaccination mice. (**B**,**C**) Graphs of ALICE clusters for p20 (**B**) and p30 vaccinated groups (**C**) with the highest enrichment in dLN over ndLN. For each cluster TRBV segment, mean frequencies within four samples and consensus CDR3 amino acid sequences are presented. If the clonotype is present in different mice and/or different lymph nodes, it is depicted by separate graph nodes. Red and green label clonotypes from dLN and ndLN, respectively. Dominant clones (if present) are also labeled with CDR3 sequences where polar amino acids are green, basic blue, acidic red, and hydrophobic amino acids are black.

The mean number of clonotypes within ALICE clusters (with an adjusted *p* value below 10^5^) was 660 ± 29 (4.9 ± 0.2%) and 946 ± 84 (8.1 ± 1.1%) for p20 and p30, indicating specific responses in both cases. Sharp differences in V-segment usage for clustered clonotypes also highlighted vaccine-specific responses (Figure 5A). The most notable differences were in TRBV17, which was in 24.4 ± 0.2% of clonotypes in p30-vaccinated mice versus 0.71 ± 0.12% in the p20 group. It speaks in favor of peptide or tumor reactivity of clonotypes with TRBV17, as this V segment usually constitutes less than 2% of the mouse repertoire [30,31]. TRBV15 and TRBV16 constitute 2.9 ± 0.5% and 6.9 ± 0.8% in the p20 group, while they are absent in the p30 group and presumably contain p20-stimulated clones. The top 30 clusters for both groups with their consensus sequences are presented in Supplementary Table S1.

To highlight tumor-specific responses, we calculated the ratios of clonotype frequencies within each cluster for dLN to ndLN (Supplementary Table S1). Three clusters having the highest dLN to ndLN ratio for each vaccine are presented in Figure 5. Then, we looked for the top five vaccine-specific clonotypes (Table 1) within all identified clusters. None of these clones have been found in ALICE clusters for the p20-vaccinated group. At the same time, the top two clones for p30 were found within ALICE clusters (Figure 5C). This difference was recapitulated if clusters were assembled with ALICE hits *p* value below 10^−4^ and without restriction to V segment (clustering only by CDR3 sequences). This indicates adaptation and convergence of the immune response in the case of top p30-specific clones.

**Figure 6:**
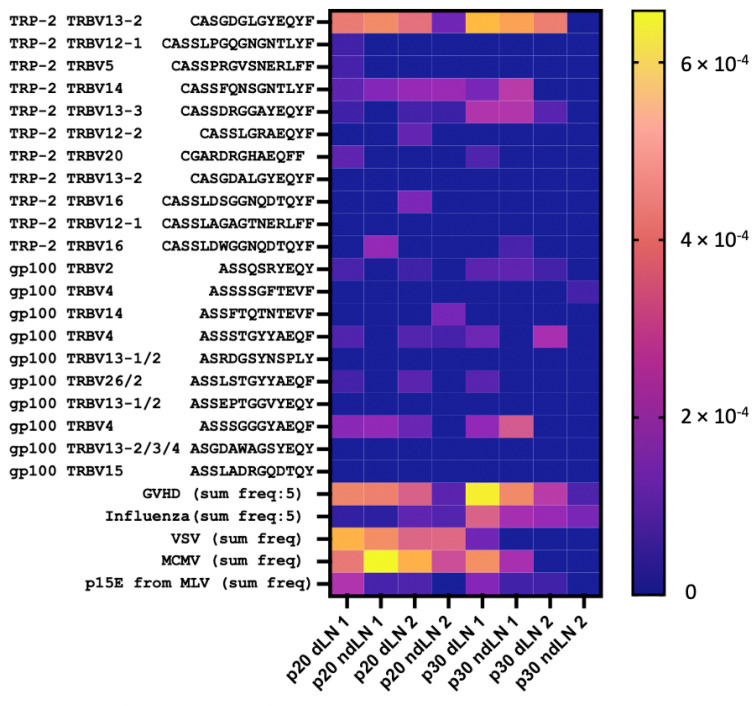
Overlap of the clonesets from the current work with the known H-2^b^-related antigen-specific clonotypes. The heatmap illustrates the frequencies of the TRP-2 and gp100 melanoma-specific clonotypes and the cumulative frequencies of GVDH-related, Influenza-, VSV-, MCMV-, and MLV-specific clonotypes within the current clonesets. Influenza and GVDH-related frequencies are divided by five for visualization. Specific clonotypes were matched with the p20 and p30 clonesets with the allowance of one amino acid mismatch and/or indel.

Vaccines were found to have different effects on the distribution of ALICE clusters between dLN and ndLN. The mean dLN/ndLN ratios for the top 30 ALICE clusters were 1.2 ± 0.11 and 0.79 ± 0.07 for p20 and p30, respectively. This means that in the case of p30-vaccinated mice, there was a significant portion of dLN-enriched clones that were not in ALICE clusters. We hypothesized that it could be caused by bystander clones that were spurred by vaccination. To test this, we analyzed our repertoires for the presence of known H-2^b^-related B16-specific clonotypes as well as autoimmune and virus-specific H-2^b^ clonotypes. We overlaid our data with the following clonotypes specific for the most immunogenic melanoma antigens: TRP-2, gp100, alloreactive H-2^b^ graft clonotypes in H-2^d^ hosts (graft versus host disease, GVHD), influenza A, vesicular stomatitis virus (VSV), mouse cytomegalovirus (MCMV), and murine leukemia virus (MLV). These clonotypes and the source data references are available in Supplementary Table S2. Many of these specific clonotypes were found in our datasets, with most frequent being TRP-2-specific (up to 0.05%), influenza-virus-specific (up to 0.13%), and GVDH-related (up to 0.1%) clonotypes (Figure 6). It is notable that with the p30 vaccination, most tumor unrelated bystander clonotypes were highly enriched in dLN, with a dLN/ndLN ratio of 2.2 ± 1.2 (mean ± SD), while with the p20 vaccine, this ratio was 1.6 ± 1.2. This allows us to hypothesize that the p30 vaccine is more likely to induce bystander activation and/or antigen spreading compared to the p20 vaccine.

## 4. Discussion

Immunosuppressive responses to peptide vaccines are not uncommon and may be due to various reasons, including the induction of Treg, Th2, or Th17 cells or the exhaustion of reactive T-cell clones [8,25,32,33,34]. Skewing towards an immunosuppressive response may be due to either adjuvant [35], antigen itself [9], or overstimulation of reactive cells [27,36]. In our study, we initially tried to minimize immunosuppressive response by using CFA that had been shown to favor Tfh response over Tfr as opposed to IFA [33,34,35,36,37,38,39]. CFA was combined with a PolyI:C adjuvant that was shown to provide good activity for antitumor vaccination in similar experimental settings [3,38]. This vaccination scheme was efficient for p30, and thus, it is unlikely that the adjuvants were responsible for the failure of p20 vaccination.

Antigen loss is another possible mechanism for tumor escape. The p20 antigen is likely to be expressed only in a subset of B16F0 cells, which is one of the possible reasons for p20 vaccine failure. However, this escape mechanism would delay tumor growth, which was not observed in our study. Another possibility is that the *Tubb3* WT variant is more aggressive, but in this case, it would replace the mutant form during culturing and/or grafting, which is also not the case. However, the possibility of a tumor eliminating targeted mutations argues for the use of vaccinations with antigens that also elicit immune reactivity for wild-type forms of the antigen.

Cell exhaustion and/or apoptosis may be another reason for unresponsiveness to tumor vaccines [36,38,40]. It was shown that CTLs are depleted at sites of antigen persistence after their administration in IFA [36]. In our experiments, we used either a short-lived formulation with water-soluble PolyI:C only or a combination of CFA for the first immunization, followed by a boost with PolyI:C. With the first scheme, we demonstrated tumor tolerance, indicating that it was developed without prolonged antigen persistence at the tumor site. With the second scheme, we compared p20 with p30 and have shown that their differences are not adjuvant-dependent. This does not exclude vaccination-dependent exhaustion or elimination but indicates that it is peptide-specific.

Cells from p20-vaccinated mice have better responsiveness to the antigen in vitro compared to p30. However, in vitro conditions discard many factors that are present in vivo, including those that may shape the immune response and impose anergy on specific clones. Metabolic regulation of Th/Treg balance, disregard of in vivo cytokine milieu and tissue microenvironment, nutrient availability, different compositions, and properties of antigen-presenting cells are among these factors. Several factors contribute to the development of tolerance in the case of p20 compared to p30. (1) Better in vitro responsiveness for p20 indicates a stronger immune response that is more likely to lead to exhaustion and tolerance. (2) For the short 10mer p20 and p30 peptides designed to bind MHC I, the predicted affinity of the p20/MHC complex was 10-times higher than for p30/MHC [41]. This agrees with the stronger response of p20 that we have seen in vitro. Prolonged exposure and better MHC binding can cause tolerance [36,42]. (3) The unmutated form of p20 antigen, tubulin beta 3 class III, is expressed predominantly in nervous tissues, while the parental p30 antigen, Kinesin family member 18B (KIF18B), is present mainly in lymphoid tissues (www.proteinatlas.org, accessed on 7 March 2024). This may lead to preformed immune memory and tolerance for unmutated p30 antigen in vitro and in vivo, with cross-reactivity to p30. This possible natural tolerance restrains p30 reactivity in vitro for LN-isolated cells by KIF18B-specific Tregs, but it is unleashed in tumors due to the absence of these Tregs.

We also observed significantly enlarged lymph nodes in peptide-vaccinated mice, while in adjuvant-only group LNs, they were also enlarged but much less. However, the most abundant were bystander cells characterized by the absence of dLN enrichment or that were found in databases as public or virus-specific TCRs. These clones may have been expanded due to adjuvant stimulation of viral-specific clones and because of the cross-reactivity of antiviral and antitumor clones [43,44].

As in a number of previous studies, we have also encountered difficulties in the detection of antigen-specific clones among high and noisy backgrounds [19,30,45,46,47]. However, the repertoire of CD8+ cells appeared to be more clonal than the repertoire of CD4+ cells [48]. Here, we dissected specific responses using several approaches. We used enrichment in dLN over ndLN as an indication of tumor reactivity. In conjunction with the analysis of TCR clustering, this appeared to be the efficient metric that highlighted differences between p20 and p30 vaccines. For validation of this pipeline, we searched consensus CDR3 sequences in available TCR data. For several dLN-enriched clones, we have found relevant variants that were either induced by anti-CTLA4 immunotherapy or allogeneic skin grafts [19,20]. This confirmed the potential antitumor reactivity of identified sequences in p30-vaccinated mice and validated our identification pipeline.

It should be noted that not all dLN-enriched clones have convergent variants; for example, the allograft skin graft-specific clone CASSDRVEQYF. Such non-convergent clones are highly enriched in dLN after p30 vaccination since clustered clones were skewed towards ndLN. Nevertheless, such individual clones are not convergent, and each individual clone accounts for less than 0.8%; if taken together, they may constitute up to 20% (according to the disbalance in the ALICE clusters). This indicates that either clustering algorithms are not efficient enough in the identification of such clones or vaccination following a tumor challenge induces a non-convergent response. The latter possibility is unlikely because it has been shown that vaccination- and tumor-induced responses are usually polyclonal with shared unrelated reactivities [45,49,50]. These reactivities stem from public clonotypes or some persistence infection- or microbiota-specific clonotypes that are usually polyclonal.

The principles of sharing or spreading immune responses are still not clear, but they appear to be an important component of an effective therapy. Both vaccines were shown to induce CD4+ T-cells primarily [2,3]. Here, we revealed that both CD4 and CD8 responses were activated for both vaccines. Moreover, CTL clonotypes with presumed tumor reactivity in the case of the p30 vaccine are quite diverse (Table 1 and Table S1), making them unlikely to target a single antigen. Such diverse specific clones may appear because of antigen spreading—the phenomenon when an antigen-presenting cell is licensed by the Th cell for activation of other T-cells, reactive to other foreign antigens presented by this cell [51]. Antigen spreading and bystander activation might be important components of an effective antitumor vaccine. The deficiency of these components in vitro does not allow for full therapeutic activity in an in vitro restimulation assay.

## 5. Conclusions

In this work, we addressed the long-lasting but still unresolved question of the low efficiency of some antitumor peptide vaccines despite their immunogenicity. We used two B16 neoantigen peptides that have been validated for immunogenicity in vivo and in vitro but have different therapeutic efficiencies. We have used deep sequencing of CTL repertoires after tumor challenge for comparison of vaccine-induced antitumor immunity. To reveal the differences between the peptides, we have used metrics based on enrichment in dLN compared to ndLN and the occurrence of similar (convergent) variants. The hallmarks of a prophylactically efficient vaccine were elevated CTL clonality in dLN and the convergence of enriched clones. It was also noted that dLN-elevated clones have different and/or shared specificities, and some of them are nonconvergent. This indicates that our method for dissecting tumor-specific responses and our knowledge of the immune mechanisms of efficient anticancer vaccination are far from exhaustive.

## Figures and Tables

**Table 1:** The top five CTL clones specifically expanded after vaccination with either the p20 or p30 peptide.

Group	TRBV	CDR3	Frequencies, × 10^−3^				Occurrence, Reference
			dLN1	ndLN1	dLN2	ndLN2	
p20	17	CASSPRQGANTEVFF	2.7	6	-	-	Public, [22]
p20	16	CASSLDDINTEVFF	-	-	3.2	2.9	
p20	14	CASSFSWGGDTQYF	-	-	2.1	4.2	
p20	20	CGALTGENTLYF	4.1	1.24	-	-	
p20	12-2	CASSLVGGYEQYF	-	0.05	3.45	1.5	Public, [23]
p30	17	CASSRRNYAEQFF	7.2	6.4	-	-	Public, enriched after a CTLA4 treatment, [19,21]
p30	29	CASSPGQANTEVFF	8.5	3.3	0.3	0.01	MC38 colon cancer CTLA4 response, [19]
p30	29	CASSPGQSAETLYF	1.35	0.8	8.3	-	Public, [22] SOX13KO, [24]
p30	15	CASSDRVEQYF	0.16	-	8.7	1	Allogenic skin graft, [20]
p30	13-2	CASGDARNTLYF	-	0.05	8.5	-	Public, [22]

## Data Availability

CTL repertoires from this work and flow cytometry data corresponding to Figure 2 are deposited on the figshare cloud: https://doi.org/10.6084/m9.figshare.24917685, and https://doi.org/10.6084/m9.figshare.25256983.

## Supplementary Materials

The following supporting information can be downloaded at: https://www.mdpi.com/2076-393X/12/4/345/s1, Figure S1: Heatmap of normalized DESeq2 log expression of target antigens (p30, *Kif18b*; p20, *Tubb3*) in B16F10 and B16F0 melanoma cells compares to housekeeping genes; Figure S2: Flow cytometry data gaiting schemes used in this study; Figure S3: ALICE clusters for p20 and p30 vaccinated groups; Table S1: Characteristics of the top 30 ALICE clusters found in p20 or p30 vaccinated mice after tumor challenge in draining (dLN) and non-draining (ndLN) lymph nodes; Table S2: Overlapping of H-2^b^-related antigen-specific clonotypes with the current clonesets. References [52,53,54,55,56,57,58,59] are cited in the Supplementary Materials.
