# Supplementary material for: Local Enrichment with Convergence of Enriched T-Cell Clones Are Hallmarks of Effective Peptide Vaccination against B16 Melanoma
Source: Vaccines (Basel). 2024 Mar 22;12(4):345. doi: 10.3390/vaccines12040345 (PMC11487401; doi:10.3390/vaccines12040345)
Supplement: Supplementary file 1 [file vaccines-12-00345-s001.zip › vaccines-2851870-supplementary.pdf]

Supplementary data for the article

Local Enrichment with Convergence of Enriched T-Cell Clones Are Hallmarks of Effective Peptide Vaccination against B16 Melanoma

Anna Vyacheslavovna Izosimova [1], Alexandra Valerievna Shabalkina [2,3], Mikhail Yurevich Myshkin [3], Elizaveta Viktorovna Shurganova [1], Daria Sergeevna Myalik [1,4], Ekaterina Olegovna Ryzhichenko [2], Alina Faritovna Samitova [5], Ekaterina Vladimirovna Barsova [2,3], Irina Aleksandrovna Shagina [2,3], Olga Vladimirovna Britanova [2,3], Diana Vladimirovna Yuzhakova [1] and George Vladimirovich Sharonov [1,2,3,*]

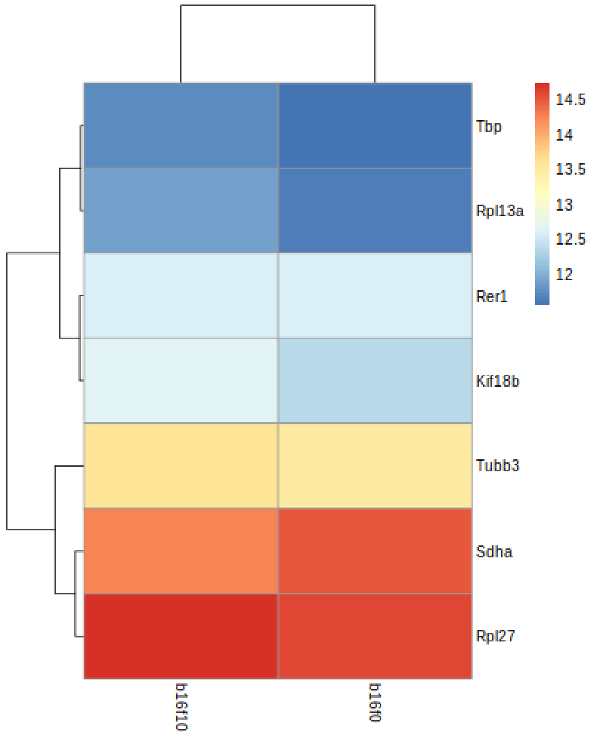

Figure S1. Heatmap of normalized DESeq2 log expression of target antigens (p30, *Kif18b*; p20, *Tubb3*) in B16F10 and B16F0 melanoma cells compares to housekeeping genes.

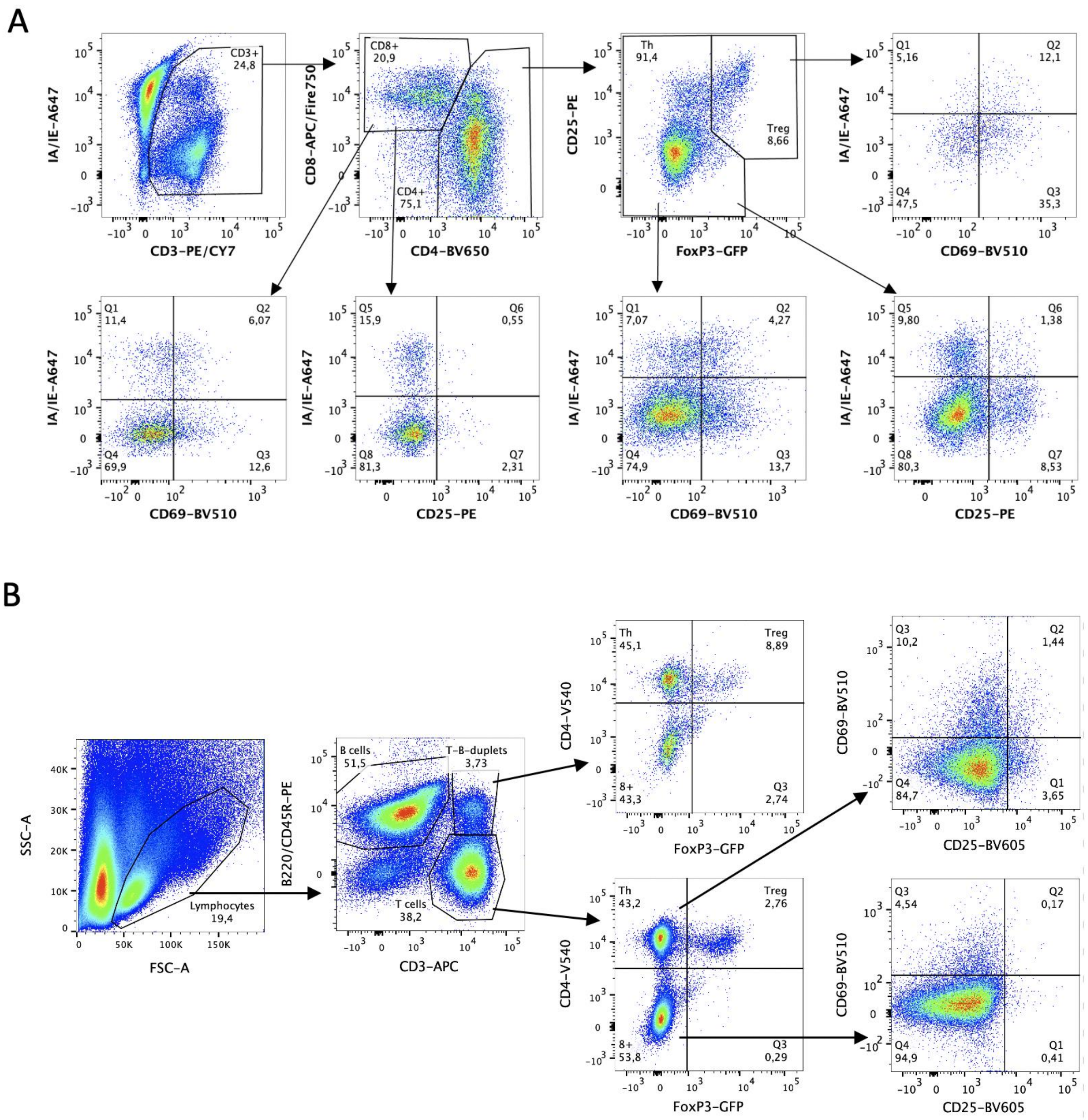

Figure S2. Flow *cytometry* data gaiting scheme used for evaluation of *in vitro* restimulated cells (A, corresponds to Figure 2) and lymph node cells from tumor bearing mice (B, corresponds to Figure 3).

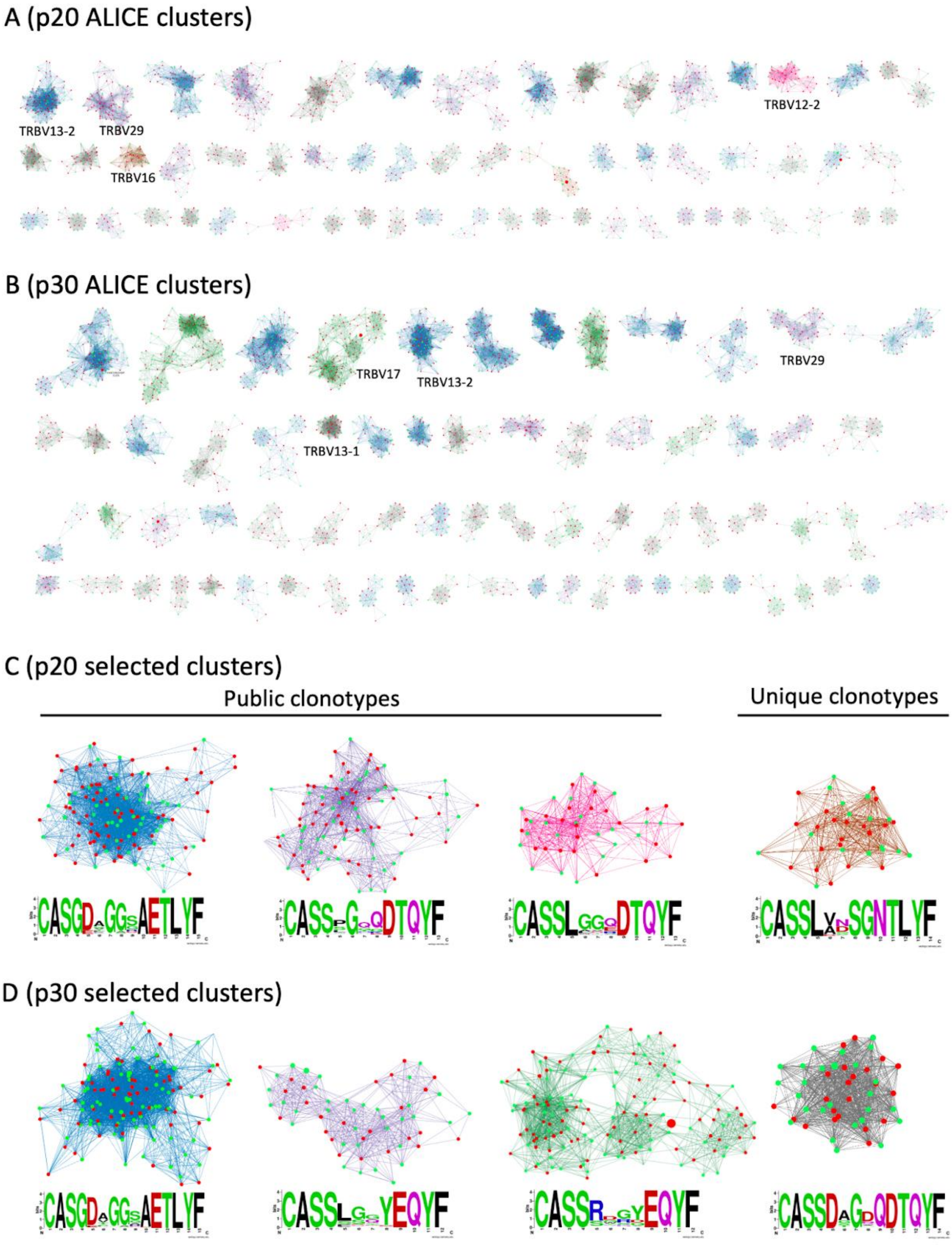

Figure S3. ALICE clusters for p20 (**A,C**) and p30 (**B,D**) vaccinated groups. Clusters are TRBV-specific. Edges of clusters with certain TRBVs are colored with specific color (TRBV13-2: blue, TRBV29: purple, TRBV12-2: magenta, TRBV16: brown, TRBV17: green) and other have gray edges. A.

All clusters with more that 10 nodes. B. Selected clusters that are labeled A with consensus CDR3 sequences are presented.

Table S1. Characteristics of top 30 ALICE clusters found in p20 or p30 vaccinated mice after tumor challenge in draining (dLN) and non-draining (ndLN) lymph nodes

**Peptide-cluster #**	**TRB V**	**Fraction % ***	**dLN/ndLN ratio**	**Consensus CDR3 motive**	**Dominant CDR3: Occurrence, Reference**
p20-1	13-2	0.83	1.27	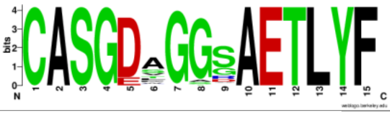	CASSGDAGGSAETLYF:p30
p20-2	29	0.82	1.07	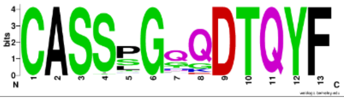	CASSPGQQDTQYF:Public [1]
p20-3	13-2	0.54	0.93	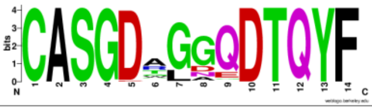	CASGDAGGQDTQYF:public [1]
p20-4	29	0.86	1	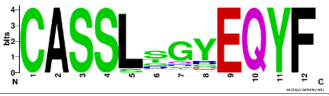	CASSLSGYEQYF:InflA$ [VDJdb]#
p20-5	13-3	0.55	1.17	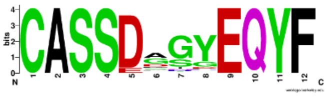	CASSDAGYEQYF: gp70 AH1 peptide, present in 4T1 tumor [2]
p20-6	13-2	0.51	1.11	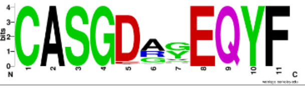	CASGDAGEQYF: public [1,3]
p20-7	29	0.6	1.25	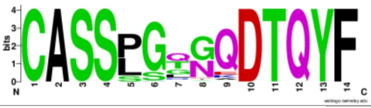	CASSPGQGQDTQYF: public [1]
p20-8	13-2	0.51	1.34	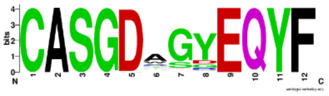	CASSDAGYEQYF: gp70 AH1 peptide, present in 4T1 tumor [2]
p20-9	12-1	0.44	0.6	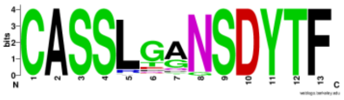	CASSLGANSDYTF: public [3], enterovirus VP1 prot. [4], vesicular stomatitis virus [VDJdb]
p20-10	13-3	0.5	0.87	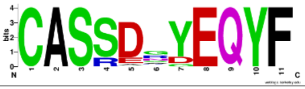	CASSDGYEQYF: public [1]
p20-11	29	0.58	0.93	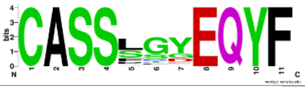	CASSLGYEQYF: InflA [VDJdb]
p20-12	13-2	0.37	0.91	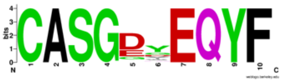	CASGDYEQYF: Public [1], diabetic mice [McPAS-TCR]$
p20-13	12-2	0.34	1.27	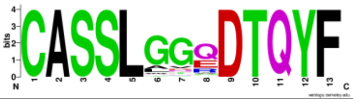	CASSLGGQDTQYF
p20-14	13-2	0.25	1.13	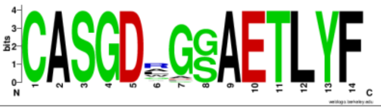	CASSDRGGAETLYF

p20-15	12-1	0.27	1	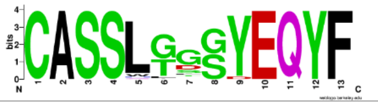	CASSLGGGYEQYF
p20-16	1	0.21	0.4	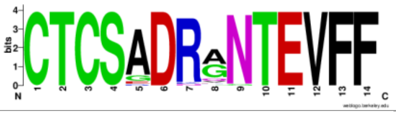	CTCSADRANTEVFF
p20-17	12-1	0.27	2.17	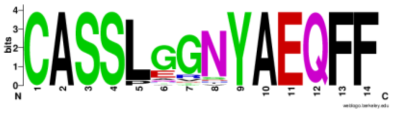	CASSLGGNYAEQFF<br>CASSLGDNYAEQFF:mCMV
p20-18	16	0.3	0.85	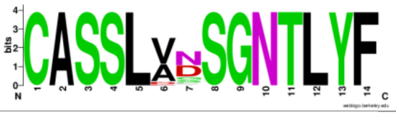	CASSLVNSGNTLYF
p20-19	29	0.25	1.6	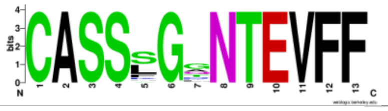	CASSSGGNTEVFF
p20-20	13-1	0.22	1.65	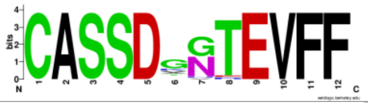	CASSDGGTEVFF
p20-21	13-1	0.2	0.6	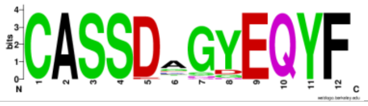	CASSDAGYEQYF
p20-22	29	0.19	1.34	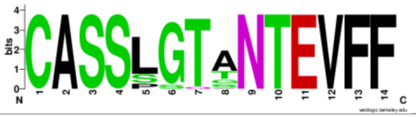	CASSLGTANTEVFF
p20-23	13-2	0.18	0.67	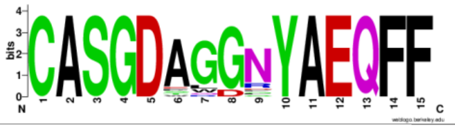	CASSDAGGNYAEQFF
p20-24	13-2	0.17	0.64	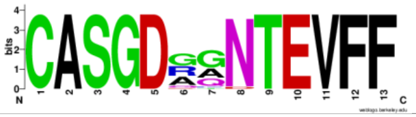	CASGDGGNTEVFF
p20-25	13-3	0.2	0.26	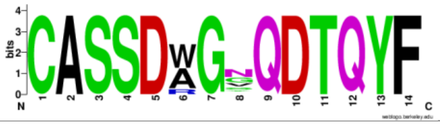	CASSDWGNQDTQYF
p20-26	19	0.18	2.33	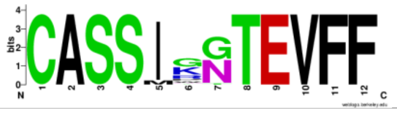	CASSIGGTEVFF<br>CASSIGRTEVFF:InflA
p20-27	16	0.34	3.17	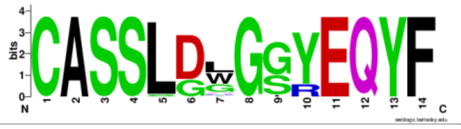	CASSLDLGGYEQYF
p20-28	13-2	0.14	0.87	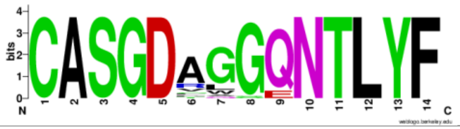	CASGDAGGQNTLYF
p20-29	13-2	0.16	1.92	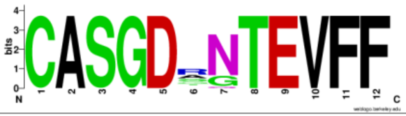	CASGDRNTEVFF
p20-30	29	0.18	1.62	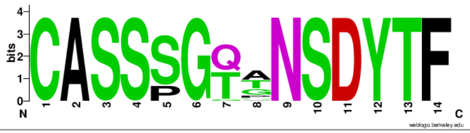	CASSSGQANSDYTF

p20-1-30		11.16	1.08		
p30-1	13-2	1.57	0.64	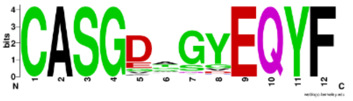	CASGDAGYEQYF<br>CASGDGGYEQYF:InflA
p30-2	17	1.5	0.73	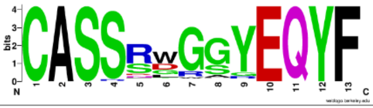	CASSRWGGYEQYF<br>CASSDWGGYEQYF: RSV
p30-3	13-2	1.02	0.67	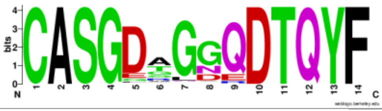	CASGDAGGQDTQYF: public [1]
p30-4	17	1.98	0.99	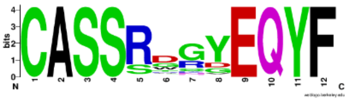	CASRDGYEQYF<br>CASRDTYEQYF: RSV [VDJdb]<br>CASRQGYEQYF: InflA [VDJdb]
p30-5	13-2	1.07	0.69	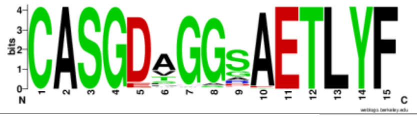	CASSGDAGGSAETLYF: p20
p30-6	13-2	0.84	0.68	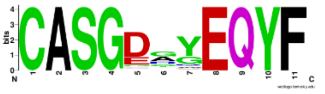	CASGDGYEQYF: auto- and cross-reactive clones to tumor and pancreas with aCTLA therapy: [5]
p30-7	13-2	0.79	0.67	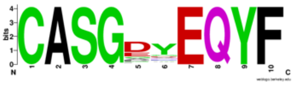	CASGDYEQYF: public [1], age-associated [6], same as for cluster p30-6
p30-8	17	0.96	0.84	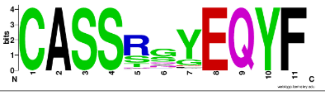	CASSRGYEQYF: public [1]
p30-9	13-2	0.5	0.65	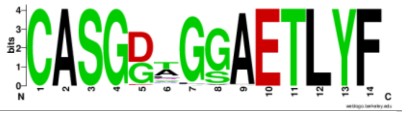	CASGDTGGAETLYF
p30-10	13-2	0.4	1	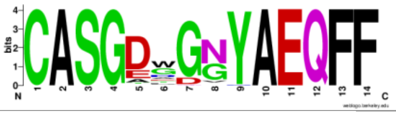	CASGDWGNAEQFF
p30-11	29	0.61	0.65	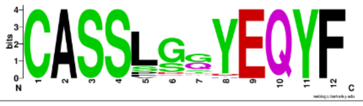	CASSLGGYEQYF: InflA
p30-12	13-2	0.39	0.42	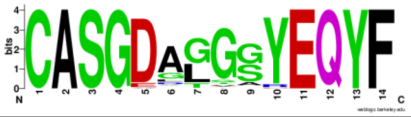	CASGDAGGGYEQYF: Myelin basic protein in MHCII [7]
p30-13	1	0.39	0.81	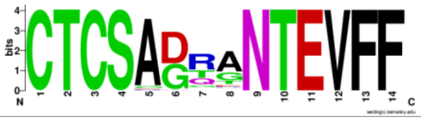	CTCSADRANTEVFF: P20<br>CTCSADRVNTEVFF: mCMV<br>CTCSADRENTEVFF: InflA
p30-14	13-2	0.38	0.5	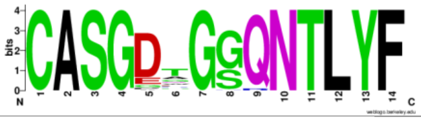	CASGDTGGQNTLYF
p30-15	13-1	0.4	0.76	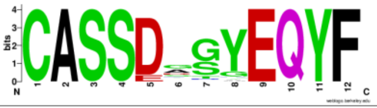	CASSDGGYEQYF

p30-16	13-2	0.36	0.76	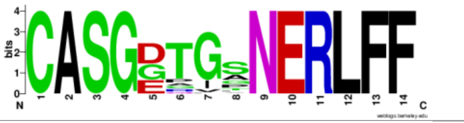	CASGDTGSNERLFF
p30-17	13-1	0.34	0.75	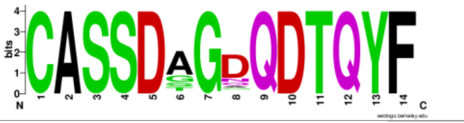	CASSDAGDQDTQYF
p30-18	13-2	0.3	0.49	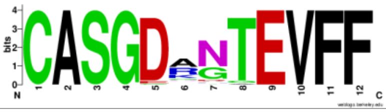	CASGDANTEVFF
p30-19	13-2	0.31	0.5	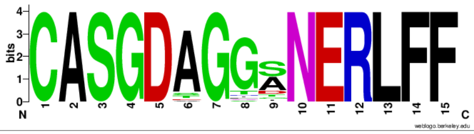	CASGDAGGSNERLFF
p30-20	13-3	0.37	0.55	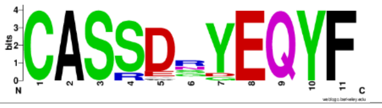	CASSDRYEQYF
p30-21	29	0.92	0.82	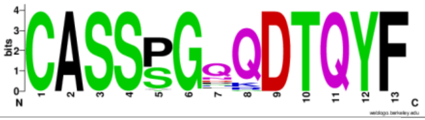	CASSPGQQDTQYF
p30-22	1	0.23	0.83	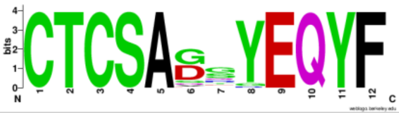	CTCSAGGYEQYF
p30-23	29	1.17	1.36	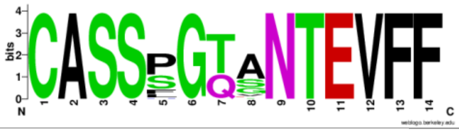	CASSPGTANTEVFF: public [1]
p30-24	17	0.3	1.14	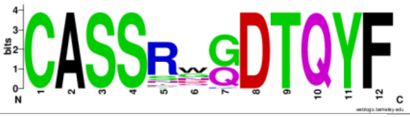	CASSRWGDTQYF
p30-25	13-2	0.25	0.48	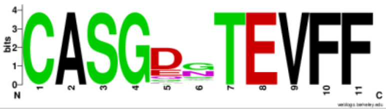	CASGDGTEVFF
p30-26	29	0.26	1.05	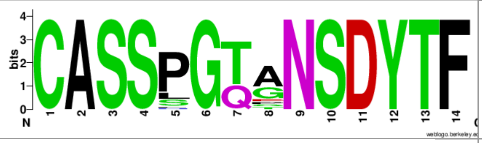	CASSPGTANSDYTF
p30-27	13-3	0.26	0.6	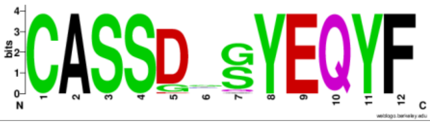	CASSD_GYEQYF
p30-28	13-2	0.14	0.74	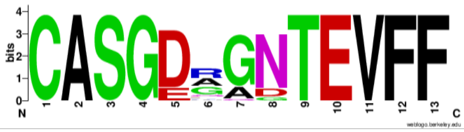	CASGDRGNTEVFF
p30-29	17	0.29	0.63	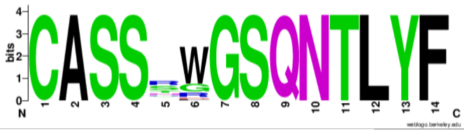	CASSRWGSQNTLYF
p30-30	29	0.44	2.36	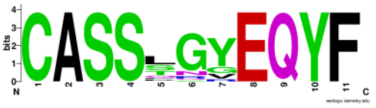	CASSLGYEQYF CASSLGGEQYF: InflA [VDJdb], public and breast cancer specific [8], enriched in CTLA KO [9]

p30-66	17	1.52	1.05	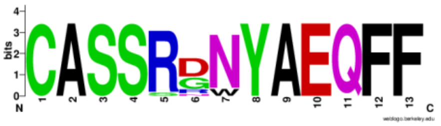	CASSRDNYAEQFF: public, enriched in aCTLA4 responders [1,10]
p30-1-30		18.84	0.77		

*Cumulative frequencies for four samples. [$]Abbreviations used: mCMV, mouse cytomegalovirus; InflA, Influenza A; RSV, Respiratory syncytial virus. [#]VDJdb database [11,12]. [$]McPAS-TCR database [13]

References

1 Kohanim, Y.K.; Tendler, A.; Mayo, A.; Friedman, N.; Alon, U. Endocrine Autoimmune Disease as a Fragility of Immune Surveillance against Hypersecreting Mutants. *Immunity* **2020**, *52*, 872–884.e5. https://doi.org/10.1016/j.immuni.2020.04.022.

2 Rudqvist, N.P.; Pilones, K.A.; Lhuillier, C.; Wennerberg, E.; Sidhom, J.-W.; Emerson, R.O.; Robins, H.S.; Schneck, J.; Formenti, S.C.; Demaria, S. Radiotherapy and CTLA-4 Blockade Shape the Tcr Repertoire of Tumor-Infiltrating t Cells. *Cancer Immunol. Res.* **2018**, *6*, 139–150. https://doi.org/10.1158/2326-6066.cir-17-0134.

3 McDonnell, W.J.; Koethe, J.R.; Mallal, S.A.; Pilkinton, M.A.; Kirabo, A.; Ameka, M.K.; Cottam, M.A.; Hasty, A.H.; Kennedy, A.J. High CD8 T-Cell Receptor Clonality and Altered CDR3 Properties Are Associated With Elevated Isolevuglandins in Adipose Tissue During Diet-Induced Obesity. *Diabetes* **2018**, *67*, 2361–2376. https://doi.org/10.2337/db18-0040.

4 Schober, K.; Fuchs, P.; Mir, J.; Hammel, M.; Fanchi, L.; Flossdorf, M.; Busch, D.H. The CMV-Specific CD8+ T Cell Response Is Dominated by Supra-Public Clonotypes with High Generation Probabilities. *Pathogens* **2020**, *9*, 650. https://doi.org/10.3390/pathogens9080650.

5 Collier, J.L.; Pauken, K.E.; Lee, C.A.A.; Patterson, D.G.; Markson, S.C.; Conway, T.S.; Fung, M.E.; France, J.A.; Mucciarone, K.N.; Lian, C.G.; et al. Single-Cell Profiling Reveals Unique Features of Diabetogenic T Cells in Anti-PD-1-Induced Type 1 Diabetes Mice. *J. Exp. Med.* **2023**, *220*, e20221920. https://doi.org/10.1084/jem.20221920.

6 Mogilenko, D.A.; Shpynov, O.; Andhey, P.S.; Arthur, L.; Swain, A.; Esaulova, E.; Brioschi, S.; Shchukina, I.; Kerndl, M.; Bambouskova, M.; et al. Comprehensive Profiling of an Aging Immune System Reveals Clonal GZMK+ CD8+ T Cells as Conserved Hallmark of Inflammaging. *Immunity* **2021**, *54*, 99–115.e12. https://doi.org/10.1016/j.immuni.2020.11.005.

7 Alli, R.; Zhang, Z.M.; Nguyen, P.; Zheng, J.J.; Geiger, T.L. Rational Design of T Cell Receptors with Enhanced Sensitivity for Antigen. *PLoS ONE* **2011**, *6*, e18027. https://doi.org/10.1371/journal.pone.0018027.

8 Gordin, M.; Philip, H.; Zilberberg, A.; Gidoni, M.; Margalit, R.; Clouser, C.; Adams, K.; Vigneault, F.; Cohen, I.R.; Yaari, G.; et al. Breast Cancer Is Marked by Specific, Public T-Cell Receptor CDR3 Regions Shared by Mice and Humans. *PLoS Comput. Biol.* **2021**, *17*, e1008486. https://doi.org/10.1371/journal.pcbi.1008486.

9 Wei, S.C.; Sharma, R.; Anang, N.-A.A.S.; Levine, J.H.; Zhao, Y.; Mancuso, J.J.; Setty, M.; Sharma, P.; Wang, J.; Pe'er, D.; et al. Negative Co-Stimulation Constrains T Cell Differentiation by Imposing Boundaries on Possible Cell States. *Immunity* **2019**, *50*, 1084–1098.e10. https://doi.org/10.1016/j.immuni.2019.03.004.

10 Philip, H.; Snir, T.; Gordin, M.; Shugay, M.; Zilberberg, A.; Efroni, S. A T Cell Repertoire Timestamp Is at the Core of Responsiveness to CTLA-4 Blockade. *Iscience* **2021**, *24*, 102100. https://doi.org/10.1016/j.isci.2021.102100.

11 Shugay, M.; Bagaev, D.V.; Turchaninova, M.A.; Bolotin, D.A.; Britanova, O.V.; Putintseva, E.V.; Pogorelyy, M.V.; Nazarov, V.I.; Zvyagin, I.V.; Kirgizova, V.I.; et al. VDJtools: Unifying Post-Analysis of T Cell Receptor Repertoires. *PLoS Comput. Biol.* **2015**, *11*, e1004503.16. https://doi.org/10.1371/journal.pcbi.1004503.

12 Bagaev, D.V.; Vroomans, R.M.A.; Samir, J.; Stervbo, U.; Rius, C.; Dolton, G.; Greenshields-Watson, A.; Attaf, M.; Egorov, E.S.; Zvyagin, I.V.; et al. VDJdb in 2019: Database Extension, New Analysis Infrastructure and a T-Cell Receptor Motif Compendium. *Nucleic Acids Res* **2019**, *48*, D1057–D1062. https://doi.org/10.1093/nar/gkz874.

13 Tickotsky, N.; Sagiv, T.; Prilusky, J.; Shifrut, E.; Friedman, N. McPAS-TCR: A Manually Curated Catalogue of Pathology-Associated T Cell Receptor Sequences. *Bioinformatics* **2017**, *33*, 2924–2929. https://doi.org/10.1093/bioinformatics/btx286.
